# IDH-Mutation Is a Weak Predictor of Long-Term Survival in Glioblastoma Patients

**DOI:** 10.1371/journal.pone.0130596

**Published:** 2015-07-09

**Authors:** Aymeric Amelot, Patricia De Cremoux, Véronique Quillien, Marc Polivka, Homa Adle-Biassette, Jacqueline Lehmann-Che, Laurence Françoise, Antoine F. Carpentier, Bernard George, Emmanuel Mandonnet, Sébastien Froelich

**Affiliations:** 1 Assistance Publique-Hôpitaux de Paris (AP-HP), Lariboisière Hospital, Department of Neurosurgery, Paris, France; 2 Assistance Publique-Hôpitaux de Paris (AP-HP), St-Louis Hospital, Department of Biochemistry, Molecular Oncology Unit, Paris, France; 3 Département de Biologie, Centre Eugène Marquis, CS 44229, Rue de la Bataille Flandres Dunkerque, 35042, Rennes Cedex, France; 4 Assistance Publique-Hôpitaux de Paris (AP-HP), Lariboisière Hospital, Department of pathology, Paris, France; 5 Assistance Publique-Hôpitaux de Paris (AP-HP), Avicennes Hospital, Department of Neurology, Bobigny, France; 6 University Paris Diderot, Sorbonne Paris Cité, Paris, France; 7 IMNC, UMR 8165, Orsay, France; University Hospital of Navarra, SPAIN

## Abstract

**Background:**

A very small proportion of patients diagnosed with glioblastoma (GBM) survive more than 3 years. Isocitrate dehydrogenase 1 or 2 (IDH1/2) mutations define a small subgroup of GBM patients with favourable prognosis. However, it remains controversial whether long-term survivors (LTS) are found among those IDH1/2 mutated patients.

**Methods:**

We retrospectively analyzed 207 GBM patients followed at Lariboisière Hospital (Paris) between 2005 and 2010. Clinical parameters were obtained from medical records. Mutations of IDH1/2 were analyzed in these patients, by immunohistochemistry for the R132H mutation of IDH1 and by high-resolution melting-curve analysis, followed by Sanger sequencing for IDH1 and IDH2 exon 4 mutations. Mutation rates in LTS and non-LTS groups were compared by Chi square Pearson test.

**Results:**

Seventeen patients with survival >3 years were identified (8.2% of the total series). The median overall survival in long-term survivors was 4.6 years. Subgroup analysis found that the median age at diagnosis was significantly higher for non long-term survivors (non-LTS) compared to LTS (60 versus 51 years, p <0.03). The difference in the rate of IDH mutation between non-LTS and LTS was statistically not significant (1.16% versus 5.9%, p = 0.144). Among LTS, 10 out of 16 tumors presented a methylation of MGMT promoter.

**Conclusions:**

This study confirms that long-term survival in GBM patients is if at all only weakly correlated to IDH-mutation.

## Introduction

Glioblastoma multiforme (GBM), which represents the highest grade of glioma, is the most common malignant primary brain tumor in adults. The median survival time for patients with GBM is about one year [1].

However, a small fraction of patients survive for more than 3 years. Close analysis of clinical, radiological, and tumor molecular characteristics of those long survivors is expected to provide some clue that would enable their identification at diagnosis. Moreover, one might discover some important marker in those patients that could be of help for designing new treatments.

Mutations in the isocitrate dehydrogenase enzyme isoform 1 (IDH1) or 2 (*IDH2*) genes have been frequently identified in gliomas. Mutations of IDH1 and IDH2 are restricted to exon 4, at codon 132 for IDH1 and codon 172 for IDH2 (hot spots). The resultants mutant enzyme gain a novel catalytic ability to produce 2-hydroxyglutarate [2]. They were demonstrated to correlate with a more favorable survival outcome [2–9].

In line with these results, a recent study evidenced a high proportion of IDH mutated tumors among long-survivors (34% versus 4.3% in controls) defined by a survival > 3 years [10]. Contrarily, a very recent study claimed that long-survivors, defined by a survival > 4 years, were not IDH-mutated [11]. The aim of this paper was to shed some light on this controversy, by comparing, within a large retrospective consecutive monocentric series, the IDH-mutation rate in long-term survivors (defined by survival > 3 years) with the rate in non long-term survivors.

## Materials and Methods

### Ethics Statement

This study was approved by local ethics committee of Pôle Neurosciences of Lariboisière Hospital. Written consent for use of molecular analysis for research purpose was obtained and recorded in each patient medical file.

### Patients

We retrospectively studied all consecutive patients with a diagnosis of intracranial GBM recorded in Lariboisière Hospital, University Medical Center of Paris 7 from 2005 to 2010. We constituted a cohort of 213 patients selected according to the criteria of histopathological diagnosis of primary GBM and age > 18 years. Tumor samples were obtained either by stereotactic biopsies or by resection. All tumors were centrally reviewed at Lariboisière Pathology Department and were classified according the World Health Organization (WHO) Classification of Tumors of the Central Nervous System [12]. Demographic information and medical data were collected using the patient’s medical charts and MRI. Data included date of birth, gender, treatment regimen, tumor grade, tumor localization, follow-up visit. Extent of surgery was assessed on post-operative MRI (when available) and operative report, and classified as stereotactic biopsy (SB), gross total resection (GTR), subtotal resection (SR), and partial resection (PR). The final status of the patient (dead or alive) could be determined based upon the register of civil status and by consulting palliative care centers or questioning the physicians, and ultimately, the close family. Among the cohort, we thus identified 17 patients with long-term survival defined by a survival of > 3 years from the diagnosis of GBM. For these 17 patients, diagnosis of GBM was confirmed by independant reviewing of histological slices.

### IDH1 Immunohistochemistry Labeling

Surgical specimens were fixed in zinc-formalin, embedded in paraffin, and routinely stained by hematoxylin-eosin-saffron (HES) and Masson trichrome. Immunohistochemical analyses were performed on paraffin embedded specimen according to the labeled avidin-biotin complex method with DAB as chromogen, using mouse monoclonal antibody against IDH1 R132H/DIA-H09 (Dianova, Hamburg, Germany), diluted at 1/70, on a Ventana Benchmark BX platform. All IDH1 immunohistochemistry labeling were performed and replayed by two separate experts.

### Detection of IDH1 and IDH2 Mutations

Tumor blocks were reviewed and selected by the pathologist to have more than 40% tumor cells. DNA was extracted from 10 μm formalin-fixed, paraffin-embedded section of primary brain tumors using an affinity column-based protocol (QIAmp DNA tissue kit, Qiagen, Courtaboeuf, France) following the manufacturer’s instructions. The quantity of isolated DNA was assessed using a NanoDrop 1000 spectrophotometer (Thermoscientific, France). A fragment of exons 4 of IDH1, spanning the R132 mutation hotspot of IDH1, and IDH2 entire exon 4 were amplified from genomic DNA by polymerase chain reaction (PCR) and mutations were screened by high-resolution melting-curve analysis (HRM) followed by direct sequencing. A 122 base pairs (bp) length fragment spanning IDH1 codons 113 to 138 was amplified using the primers previously published by Loussouarn [13]. A fragment of 290 bp length fragment spanning IDH2 entire exon 4 was amplified using the primers previously published by Frenel et al. [14]. HRM analysis was performed using HRM guidance, on a Light Cycler 480 (Roche, Diagnostics, Meylan, France). Fluorescent melting curves were evaluated using Light Cycler 480 software release 1.5.0 SP4 (Roche). After HRM, samples were purified and sequenced bidirectionally, using the same primers and the Big Dye Terminator v1.1 sequencing kit (Applied Biosystems, by Life Technologies). All sequences were analyzed for somatic mutations using seqscape software (Applied Biosystems).

### Determination of Methylation Status of MGMT Promoter

Pyrosequencing of five CpG sites from MGMT promoter was performed with the PyroMark Q24 CpG MGMT kit (ref. 970032) (Qiagen, France) on a PyroMarkQ24 system (Qiagen), as per manufacturer's recommendation. When the methylation percentage of MGMT (ie the mean value of the five CpG sites tested) was above 8%, the sample was classified as methylated [15,16].

### Statistical Analysis

Statistical analyses were performed using STATA version 11 (StataCorp, Texas, USA). Associations between different group parameters or patients group were analyzed by Fisher’s exact test. Chi-square test for variance in a normal population were performed to study the independence of the parameters and Fisher’s exact test were applied when the samples were too small. Finally, the Cox proportional model was used in a multivariate analysis of the gender, initial clinical symptomatology and extent of surgery as categorical variables, and age and Karnofsky performance status (KPS) as continuous variables. All tests were two-sided, p-values less than 0.05 were considered significant.

## Results

### Clinical Factors

Among the 213 GBM, six patients were lost to follow-up right after surgery and were subsequently excluded from the study. The final cohort included 207 patients. A total of 17 patients fulfilled inclusion criteria of survival > 3 years from diagnosis, constituting the long-term survivor (LTS) group. Among them, sex ratio was close to 1 (male 9; female 8). Four patients had right hemispheric tumors, eleven had left hemispheric tumors, while two patients had bilateral tumors. Three patients were diagnosed by biopsy alone, while the remaining had open craniotomies and tumor resection. Clinical symptomatology at the initial discovery was headache (30%), periods of focal or cognitive deficit (30%), episodes of intracranial hypertension (25%), occasionally caused by episodes of epilepsy (15%), and rarer by asthenia (5%) (Table 1). The other 190 patients were defined as non long-term survivors. Clinical characteristics are detailed in Table 2.

**Table 1:** Long-term Survivors of Malignant Glioma.

N°	Age	Dx^a^	Sex	Tumor^b^ location	KPS	Initial^C^ clinic	Initial^d^ surgery	Recidive Treatment^e^ following Initial Stupp			outcome	(years)
								2^nd^ Surgery	2^nd^ Line	3^rd^ Line		
1	55	GBM	M	L T	70	Deficit F	GTR	+	BCNU	-	Alive +R	7.9
2	46	GBM	M	L T	90	Headache	GTR	-	-	-	Dead	3,8
3	49	GBM	F	R F	90	Epilepsy	GTR	-	CA	-	Dead	6
4	61	GBM	F	R F	90	IH	PR	-	CA	BCNU	Dead	4,5
5	26	GBM	F	R P	100	Headache	GTR	-	-	-	Alive-R	6,5
6	39	GBM	F	L FT	100	Epilepsy	GTR	-	CA	-	Dead	3,5
7	57	GBM	F	L T	100	Epilepsy	GTR	-	-	-	Alive-R	7
8	73	GBM	M	CC bF	60	Deficit C,F	Sbx	-	-	-	Dead	4
9	65	GBM	M	L T	100	Deficit F	PR	-	CA	-	Dead	3,1
10	47	GBM	M	R FT	70	IH, astheny	GTR	+	BCNU	CA	Dead	3,5
11	35	GBM	F	L FP	100	Epilepsy	GTR	-	-	-	Alive-R	4,5
12	65	GBM	F	L T	90	Headache	GTR	-	-	-	Alive-R	4
13	37	GBM	M	L FP	90	Deficit C	GTR	-	-	-	Alive-R	3,7
14	51	GBM	M	L PTO	90	Deficit C	Sbx	-	-	-	Alive-R	4,1
15	52	GBM	F	L F	90	Deficit C, IH	GTR	+	Sutent	CCNU	Alive +R	4
16	62	GBM	M	bF	80	IH	Sbx	-	-	-	Alive +LF	4
17	49	GBM	M	L FP	60	Headache	GTR	-	-	-	Alive-R	3,7

^a^GBM, glioblastoma multiforme;

^b^L, left; R, right; F, frontal; T, temporal; P, parietal; O, occipital; CC, corps calleux; bF, bi-frontal

^c^IH, intracranial hypertension, Deficit focal: F, cognitive: C

^d^GTR, gross total resection; PR, partial resection; Sbx, stereotactic biopsy

^e^Stupp, radiotherapy + temozolomide; Te, Temozolomide; CA, Campto (irinotécan) and Avastin (bévacizumab); BCNU, 1,3-bis(2-chloroethyl)-1-nitrosourea; ISOPS I, immunostimulatory oligonucleotides (CpG-ODN) for recurrence GBM; CCNU, Lomustine (BELUSTINE); Sutent, sunitinib

KPS, Karnofsky Performance score

Alive +/-R; Alive with or without recidive

Alive +LF; Alive but Lost to Follow-up

**Table 2:** Clinical characteristics of long-term and non long-term survivors of glioblastoma.

		Long-term survivors	Non long-term survivors	p value
**Total population**		n = 17 (8.2%)	n = 190 (92.8%)	
	Male	n = 9 (53%)	n = 114 (54.6%)	*0*.*07*
	Female	n = 8	n = 76	
**Median age at diagnosis**		51 (26–73) years	60 (21–88) years	*< 0*.*03*
**Median KPS**		86.1 (60–100)	85.8 (40–100)	*0*.*92*
**Median Survival**		4.6 (3.1–7.9) years	0.87 (0.01–2.8) years	*< 0*.*0001*
**Tumor localization**				
	Right hemisphere	4 (23.6)	92 (48.1)	*0*.*157*
	Left hemisphere	11 (64.7)	87 (46.1)	
	Both hemisphere	2 (11,6)	11 (5.8)	
**Main lobe localization**				
	Frontal	6 (35.3)	70 (36.8)	*0*.*884*
	Temporal	6 (35.3)	65 (34.7)	
	Parietal	4 (23.6)	52(26.9)	
	Occipital	1 (5.8)	3 (1.6)	
**Primary treatment**				
	Surgery	14 (82.0)	115 (60.5)	*0*.*04*
	Stereotactic biopsy	3 (18.0)	75 (39.5)	*0*.*04*

After undergoing surgery either for biopsy or resection, all the patients were entered into “Stupp” radiochemotherapy protocols, consisting in concomitant chemo-radiotherapy with 6 months adjuvant Temozolomide. Among long-survivors, 7 patients (41%) underwent second-line chemotherapy at recurrence (see Table 1). Among the 10 long-survivors patients who did not benefit from second-line treatment, 8 had not recurred at time of last follow-up, 1 died of another cause, and 1 refused to be treated at the time of recurrence.

The median age at diagnosis in the LTS group was 51 years (range 26–73) vs 60 years (21–88) in short-term survival (non-LTS) group (p < 0.03). The median survival time was 4.6 years in the LTS group (SD = 1.4, range [3.1–7.9]) while it was 0.87 years for the non-LTS group (SD = 0.7, range [0.01–2.8, p > 0.001). Median KPS at diagnosis was 86.1 (SD = 13.3, range [60–100]) in the LTS group and was not significantly different from the median KPS in the non-LTS group (85.8, SD = 11.4, range [40–100], p = 0.92) (Table 1). The distribution of lesions in different brain lobes was similar in the two groups (p = 0.88). Furthermore, lesions were rather in the left hemisphere in the LTS group, but this was not significant (p = 0.16). Predictably, we found that the LTS group had benefited more surgical resections than others (p = 0.04).

The multivariate analysis showed that neither age (p< 0.088, IC 95% [0.959–1.820]), nor gender (p< 0.726, IC 95% [0.701–1.280]), and nor the KPS (p< 0.075, IC 95% [0.665–1.12]) were associated with increased survival. There was the same lack of significance with initial clinical symptomatology factor. Conversely, the extent of surgery was the only factor associated with increased survival: so will be in the non-LTS group, successively 0.8 years for stereotactic biopsy (SB), 1 years in partial resection (PR), 1.6 years for subtotal resection (SR) and 1.7 years for gross total resection (GTR) or even in the LTS group 3.9 years for SB, 3.07 years for PR, 3.8 years for SR and 6.6 years for GTR (95% CI [1.26–1.78], p < 0.0001 according Log Rank test).

### Biological Factors

**Fig 1:**
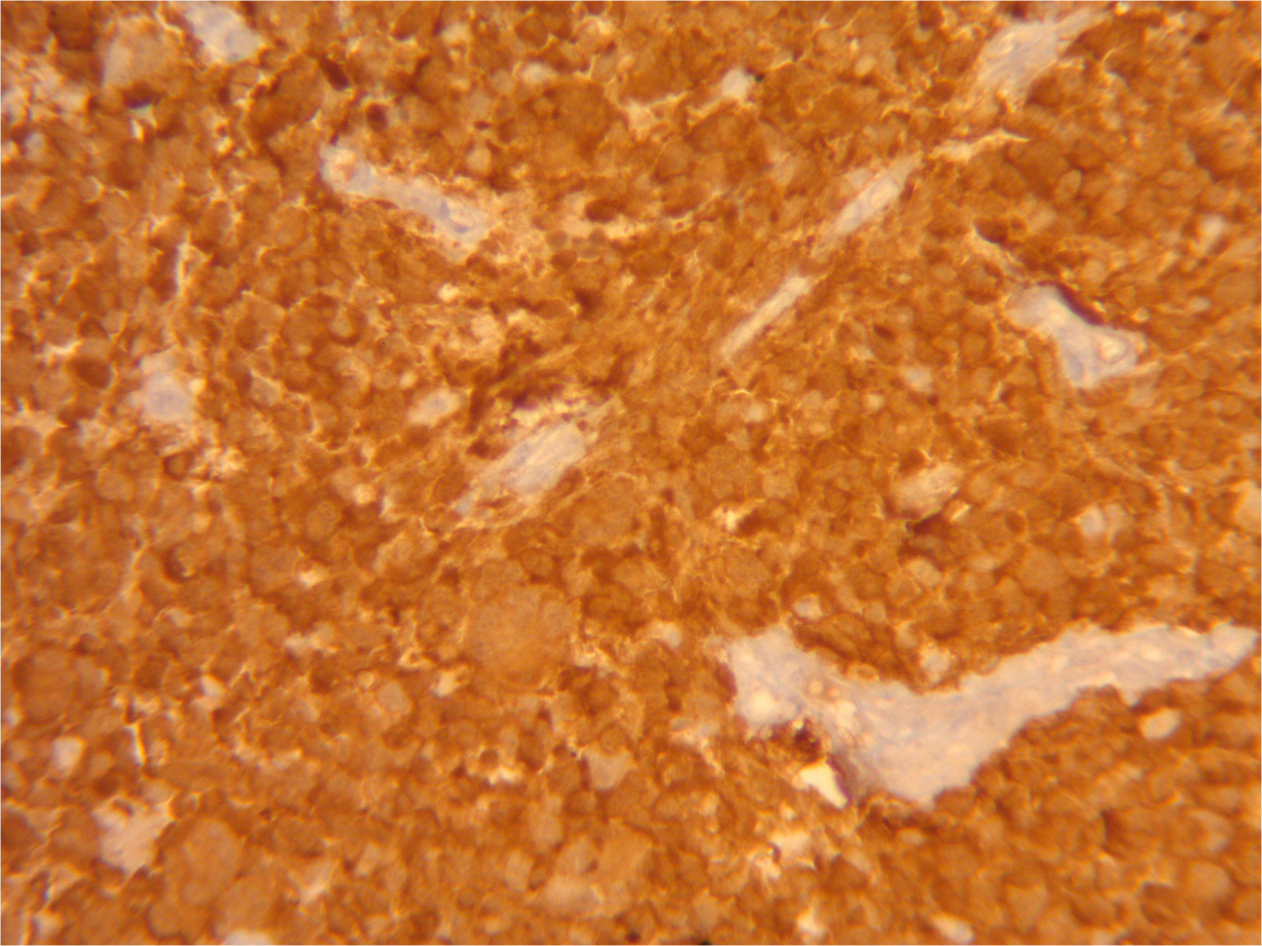
IDH1 immunohistochemistry in patient 6. Strong immunolabelling of 100% of tumor cells; negativity on vessels.

As a first step, we determined the presence of the hotspot R132H IDH1 mutation by immunohistochemistry. Surprisingly, expression was found positive for only one patient (patient 6, see Fig 1) and doubtful for an other one (patient 11), as reported in Table 3.

**Table 3:** Long-term Survivors of Malignant Glioma IDH mutations.

			IDH1 status		IDH2 status	
*Patient*	Age	Gender	Immuno-histochemistry	Molecular analysis	Molecular analysis	Survival (years)
1	55	M	-	WT	WT	7.9
2	46	M	-	WT	WT	3,8
3	49	F	-	WT	***IM***	6
4	61	F	-	WT	WT	4,5
5	26	F	-	NA	NA	6,5
6	39	F	+++	***p*.*R132H***	WT	3,5
7	57	F	-	WT	WT	7
8	73	M	-	WT	WT	4
9	65	M	-	WT	WT	3,1
10	47	M	-	WT	WT	3,5
11	35	F	+	WT	WT	4,5
12	65	F	-	WT	WT	4
13	37	M	-	WT	WT	3,7
14	51	M	-	WT	WT	4,1
15	52	F	-	WT	WT	4
16	62	M	-	WT	WT	4
17	49	M	-	WT	WT	3,7

WT: Wild Type, mR132H: mutation affected the amino acid arginine in position 132 of the amino acid sequence substituting by Histidine, IQ: Insufficient quantity

The second step was to confirm this finding and to enlarge the exploration to the other mutations of IDH1 and IDH2 on exon 4 by molecular biology, using HRM analysis followed by Sanger sequencing. DNA analyses did not reveal the presence of other mutations and confirmed the immunohistochemical detection of only one IDH1 R132H-mutated case. Thus on all 17 long survival patients, 15 (88.2%) were found IDH1 wild type (WT), only one patient (5.9%) carried the mutation, and analysis could not be performed in another one (patient 5). Regarding IDH2 gene, 15 patients are WT and two patients were undetermined because of a lack of material (patient 5) or because of poor DNA amplification (patient 3). The patient carrying the IDH1 mutation had a survival of 3.5 years, slightly lower than the median survival of LTS group (4.6 years, range 3.1–7.9 years).

We also analyzed the frequency of IDH1/2 mutations in the short-term survivors group (see Table 4.) Out of the 190 cases in this group, mutation screening was not possible in 18 cases, either because of lack of tumor samples or because of poor quality of DNA amplification, thus leaving 172 cases for subsequent testing. Only 2 patients presented the IDH1 R132H mutation. No patient had an IDH2 mutation. The difference of IDH1 mutation rate between long-term survivors and short-term survivors (5.9% versus 1.16%) was statistically not significant (p = 0.144). Similarly, the difference of long-term survivors percentage between the IDH1-mutated cohort (1/3) and the IDH1-wild type (16/186) cohort (33.3% versus 8.6%) was statistically not significant (p = 0.247).

**Table 4:** IDH1/2 mutations in long-term and non long-term survivors of patients with primary glioblastoma.

	Total Population	Median Survival	Molecular analyses	IDH1 mutations (%)	*p value*
LTS	17	4.6 years	17	1 (5.9%)	*p = 0*.*144*
nLTS	190	0.87 years	172	2 (1.16%)	

Finally, we also studied the methylation of MGMT promoter, as it has been shown in previous studies that methylation was much more frequently found in long-term survivors than in general glioblastoma population. The MGMT promoter was determined in the LTS group by pyrosequencing of five CpG sites from MGMT promoter. Methylation was observed in 10 out of 16 patients (62.5%).

## Discussion

To date, despite improvement in treatments modalities–including surgery, radiotherapy and chemotherapy–, the prognosis of glioblastoma patients remains poor, with a median overall survival < one year. There is some inconsistency in the literature regarding the definition of a long-survivor. Previous studies have been using varying time periods, ranging from 1.5 to 5 years [17,18]. In 1992, Vertosck's group has reported a series of 22 (5%) long-term survivors more than 4 years after diagnosis among a series of 556 GBM patients diagnosed and followed at the Western Pennsylvania Hospital in Pittsburgh [19]. In 2003, McLendon et al. identified a series of 32 (4.2%) very-long-term survivors (> 5 years) in a series of 766 GBM patients conducted by the Duke University Medical Center [20]. Owing to recent advances in available therapies, resulting in larger proportions of patients living longer, a three-years period is currently the most used definition of long survivors [21]. Long-term survival > 3 years with GBM is rare and is only seen in 3–10% of patients [22,23]. Old series demonstrated that only 2.2% of patients are estimated to survive 3 years or more after diagnosis [24]. A German study reported clinical and molecular analysis of 55 primary glioblastoma long-term survivors (> 3 years) at the six clinical centers of the German Glioma Network [22]. This represents one of the largest series of long-term survivors to be described to date [10]. More recently, Gerber & al. presented a series of 7 patients with survivals > 4 years, for which they characterized the rates of MGMT promoter methylation and IDH1/IDH2 mutations [11].

Among the 207 GBM patients operated on in Lariboisière's hospital between 2005 and 2010, 17 (8.2%) survived more than 3 years. The median survival of 0.875 years for the non-LTS group is in agreement with the other previously published retrospective series [21,25]. The number of long-term survivors is consistent with previously reported results [10]

Several small studies have identified clinical and tumor characteristics and treatment-related prognostic factors [26,27]. Clinical factors include age [28], race [29], Karnofsky performance status (KPS) [22], extent of surgical resection [27,30], tumor localization [31], and post-operative chemo-radiotherapy [32]. To date, among the molecular prognostic factors, only methylation of the O-6-methylguanine-DNA methyltransferase (MGMT) promoter has been found as predictive marker of GBMs' response to treatment [33]. Moreover, several studies reported a favorable impact of the IDH1/2 mutation in GBM patients [34].

Our work demonstrate that long-term GBM survivors were significantly younger than the others (51 *vs* 60, p<0.03), as previously reported [24,35]. A slight preponderance of females has been reported among long-term survivors [23]. The proportion of males and females in our LTS series was nearly 50%, although a larger number of males was non-significantly found in the non-LTS group (114 *vs* 76, p = 0.07). Nevertheless, taking into account published datas, it seems that glioblastoma long-term survival is favored by the combination of two basic clinical parameters, young age and female gender.

Previous studies have also reported that a higher preoperative KPS is associated with longer survival [22]. In contrast, we found that there was no significant difference in the preoperative KPS between the LTS and non-LTS groups: 86.1 *vs* 85.8 (p = 0.92). This difference may result from the fact that some patients with very poor KPS might have been excluded from the present series, since a stereotactic biopsy is not always performed in those patients.

Recent data have confirmed that the extent of resection is associated with improved progression-free survival [36,37]. This finding was confirmed in our study, although it is striking to note that a significant proportion (3/17 = 18%) of LTS patients only had stereotactic biopsies.

**Fig 2:**
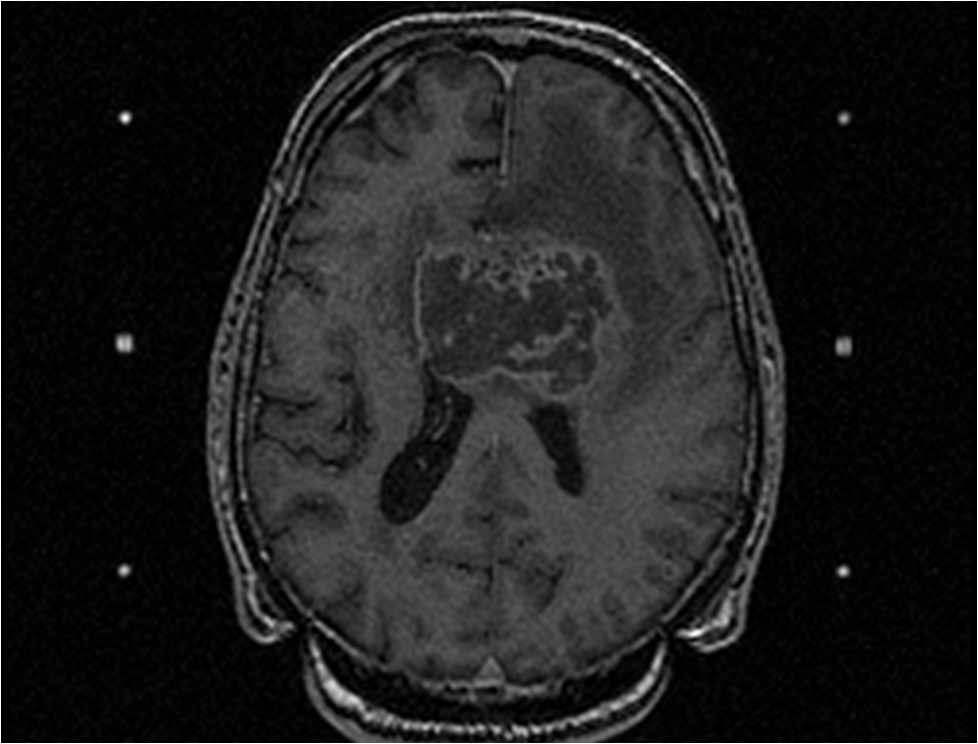
MRI performed for a seventy-three-year-old man presented to the emergency department with bilateral muscular weakness members, cognitive disorders and KPS of 60. The T1W sequence with gadolinium injection showed a large bifrontal GBM invading the corpus callosum. Stereotactic biopsy revealed the presence of a glioblastoma multiforme (confirmed by independent reviewing). This patient had almost all factors of poor prognosis (sex, age, low KPS, tumor localization, no surgical treatment) but received concomittant radiochemotherapy with temozolomide and survived 4 years.

All long-term survivors had adjuvant radiochemotherapy according to the Stupp Protocol. Therefore, these data are in support of a positive role for chemotherapy in glioblastoma with respect to long-term survival [38], even when resection is not possible (see illustrative case in Fig 2).

As we failed to find meaningful way of real predictors of good prognosis in the present series of long survivors, we evaluated the impact of *IDH* mutational status on outcomes. Isocitrate dehydrogenase (IDH), whose activities are dependent on either nicotinamide adenine dinucleotide phosphate (IDH1 and IDH2) or nicotinamide adenine dinucleotide (IDH3), catalyzes the oxidative decarboxylation of isocitrate (ICT) to produce a-ketoglutarate (α-KG). *IDH1* mutations were initially discovered in a subset of GBMs by large-scale sequencing [39]. All of the observed alterations are somatic, heterozygous mutations and occur at highly conserved positions. It has been reported that *IDH1* mutations represent an early event in tumorigenesis. It is an independent favorable prognostic marker in human gliomas [6].

The last years were marked by advances on the possible prognosis role of the IDH1/2 mutation in GBM. The German Glioma Network performed genome and transcriptome-wide molecular profiling of glioblastoma samples from 28 long-term survivors with >3 years overall survival and finally concluded their work finding that IDH1/2 mutation was associated with distinct genomic and transcriptomic changes that together define a molecular subtype of glioblastoma with better prognosis and increased likelihood for long-term survival [10,40]. Their results are similar to those provided by an analysis of the TCGA dataset [11], with 27% of IDH mutations in 22 LTS, versus 4% in non-LTS. More recently, and, poles apart, Gerber et al concluded from their series that while IDH mutant proneural tumors impart a better prognosis in the short-term, survival beyond 4 years does not require IDH mutation and is not dictated by a single transcriptional subclass [11]. This finding is in complete agreement with our study, since very surprisingly, only 1 patient is IDH1 mutated in the group of 17 long-term survivors. Interestingly, when comparing IDH1 mutation related to age, we found that the median age of IHD1 mutated patients were not significantly younger than the IDH1 WT: 46.3 vs 59.8 years (p = .0.08).

Our results are in line with those of the MSKCC series, and somehow different from those of the German and TCGA series. We cannot rule out that a lack of statistical power of the present study could explain this discrepancy and that statistical significance would be reached by increasing the size of the series. However, we rather make ours the hypothesis of Gerber & al. of “a selection bias or a difference in the biological distribution of the patients”. Indeed the percentage of IDH-mutated malignant glioma in the German series (10%) is much higher than in our series (1.6%). The well known variability of histopathological diagnosis might explain this difference.

Since the introduction of temozolomide (TMZ) chemotherapy in the standard care protocol for glioblastoma (GBM) patients, everyone agrees that the analysis of O6-methylguanine DNA methyltransferase (MGMT) status has become a key biological marker [33,41,42]. Molenaar et al have recently concluded on their series of long survivors that the combination of IDH1 mutations and MGMT methylation outperforms either IDH1 mutations or MGMT methylation alone in predicting survival of glioblastoma patients [43]. The high rate (62.5%) of methylation of MGMT promoter in our LTS series is in line with previous reports (77.8% in [44], 60% in [10] 71% in [11], and 67% in the TCGA LTS cohort, as reported in [11]). In comparison, reviewing the literature for the rate of methylation of MGMT promoter in non-LTS series yields a value around 45% [45]

## Conclusion

In conclusion, we described in the present study a large cohort of 207 GBM patients treated in Lariboisière Hospital, of whom 17 were found to be long-term survivors. In agreement with the data literature and previous studies, age at diagnosis, gender, and treatment (surgery vs biposy) are obvious prognostic factors. MGMT promoter was found to be frequently methylated. In contrast, we could not demonstrate a higher rate of *IDH1/2* mutation in the long-term survival group, highlighting the need to identify other prognostic factors. This might be achieved by evaluating some imaging parameters, including velocity of tumor growth before any treatment, or by performing further molecular analysis.
